# Chest dual-energy CT to assess the effects of steroids on lung function in severe COVID-19 patients

**DOI:** 10.1186/s13054-022-04200-z

**Published:** 2022-10-25

**Authors:** Gaetano Perchiazzi, Aleksandra Larina, Tomas Hansen, Robert Frithiof, Michael Hultström, Miklos Lipcsey, Mariangela Pellegrini

**Affiliations:** 1 Anesthesiology and Intensive Care Medicine, Department of Surgical Sciences, Uppsala University, Uppsala, Sweden; 2 Hedenstierna Laboratory, Department of Surgical Sciences, Uppsala University, Akademiska Sjukhuset, Ing 40, 3 tr, 751 85 Uppsala, Sweden; 3 Department of Anesthesia, Operation and Intensive Care, Uppsala University Hospital, Uppsala, Sweden; 4 Section of Radiology, Department of Surgical Sciences, Uppsala University, Uppsala, Sweden; 5 Integrative Physiology, Department of Medical Cell Biology, Uppsala University, Uppsala, Sweden

**Keywords:** COVID-19, Dual-energy CT, Steroids, Hypoxic pulmonary vasoconstriction

## Abstract

**Background:**

Steroids have been shown to reduce inflammation, hypoxic pulmonary vasoconstriction (HPV) and lung edema. Based on evidence from clinical trials, steroids are widely used in severe COVID-19. However, the effects of steroids on pulmonary gas volume and blood volume in this group of patients are unexplored.

**Objective:**

Profiting by dual-energy computed tomography (DECT), we investigated the relationship between the use of steroids in COVID-19 and distribution of blood volume as an index of impaired HPV. We also investigated whether the use of steroids influences lung weight, as index of lung edema, and how it affects gas distribution.

**Methods:**

Severe COVID-19 patients included in a single-center prospective observational study at the intensive care unit at Uppsala University Hospital who had undergone DECT were enrolled in the current study. Patients’ cohort was divided into two groups depending on the administration of steroids. From each patient’s DECT, 20 gas volume maps and the corresponding 20 blood volume maps, evenly distributed along the cranial–caudal axis, were analyzed. As a proxy for HPV, pulmonary blood volume distribution was analyzed in both the whole lung and the hypoinflated areas. Total lung weight, index of lung edema, was estimated.

**Results:**

Sixty patients were analyzed, whereof 43 received steroids. Patients not exposed to steroids showed a more extensive non-perfused area (19% vs 13%, *p* < 0.01) and less homogeneous pulmonary blood volume of hypoinflated areas (kurtosis: 1.91 vs 2.69, *p* < 0.01), suggesting a preserved HPV compared to patients treated with steroids. Moreover, patients exposed to steroids showed a significantly lower lung weight (953 gr vs 1140 gr, *p* = 0.01). A reduction in alveolar–arterial difference of oxygen followed the treatment with steroids (322 ± 106 mmHg at admission vs 267 ± 99 mmHg at DECT, *p* = 0.04).

**Conclusions:**

The use of steroids might cause impaired HPV and might reduce lung edema in severe COVID-19. This is consistent with previous findings in other diseases. Moreover, a reduced lung weight, as index of decreased lung edema, and a more homogeneous distribution of gas within the lung were shown in patients treated with steroids.

*Trial registration*: Clinical Trials ID: NCT04316884, Registered March 13, 2020.

**Supplementary Information:**

The online version contains supplementary material available at 10.1186/s13054-022-04200-z.

## Background

The spread of the COVID-19, caused by the beta coronavirus SARS-CoV-2, led to the declaration of a pandemic in March 2020 [1]. Since then, an “unprecedented flood of research on the coronavirus” [2] brought to the elucidation of many molecular mechanisms subtending the disease [3–5]. However, despite the prompt recognition of the consequences of SARS-CoV-2 on different organs and functions [6], the pathophysiological mechanisms affecting the respiratory function still remain to be thoroughly understood. This difficulty derives from the fact that the primary function of the respiratory system, i.e., the gas exchange, is based on the interplay of two different sub-systems [7], alveolar ventilation and pulmonary capillary perfusion that deal with different physiological mechanisms and are associated with different structural features.

Numerous drugs have been tested as anti-SARS-CoV-2 agents [8]. Among them, steroids (i.e., dexamethasone 6 mg for 10 days) have been demonstrated to improve survival [9, 10] and are therefore widely used in severe COVID-19. Despite their effectiveness and well-known pleiotropic effects [11], the pharmacodynamics of steroids on COVID-19 lungs have not yet been widely reported. Based on the previous literature, a steroid-induced dysregulation of hypoxic pulmonary vasoconstriction (HPV) [12, 13] as well as a reduction in lung edema [14] can be hypothesized to occur in COVID-19 patients.

Already in the early stages of the pandemic, lung perfusion abnormalities were reported based on dual-energy computed tomography (DECT) [15]. Contrast-enhanced DECT is a powerful lung imaging method based on two concurrent X-ray energy spectra. Taking advantage of the different attenuation profiles of different substances, including iodine contrast, DECT can *simultaneously* characterize regional distribution of gas and blood volume in the lung parenchyma [16, 17].

The main objective of this study was to investigate the effects of steroids on regional distribution of pulmonary gas and blood volume as indices of shunting affecting oxygenation in critically ill patients with severe COVID-19 using data from contrast-enhanced DECT.

The study had three main aims: firstly, to examine the possibility of visualize pharmacodynamical mechanisms of drugs on lung parenchyma using DECT; secondly, to investigate the association between the use of steroids and HPV in COVID-19 lungs and therefore examine the possible influence of steroids on ventilation/perfusion matching; and thirdly, to explore the association between steroids therapy and magnitude of lung edema and consequently the effects on regional distribution of gas volume and oxygenation.

## Methods

### Study population

**Fig. 1:**
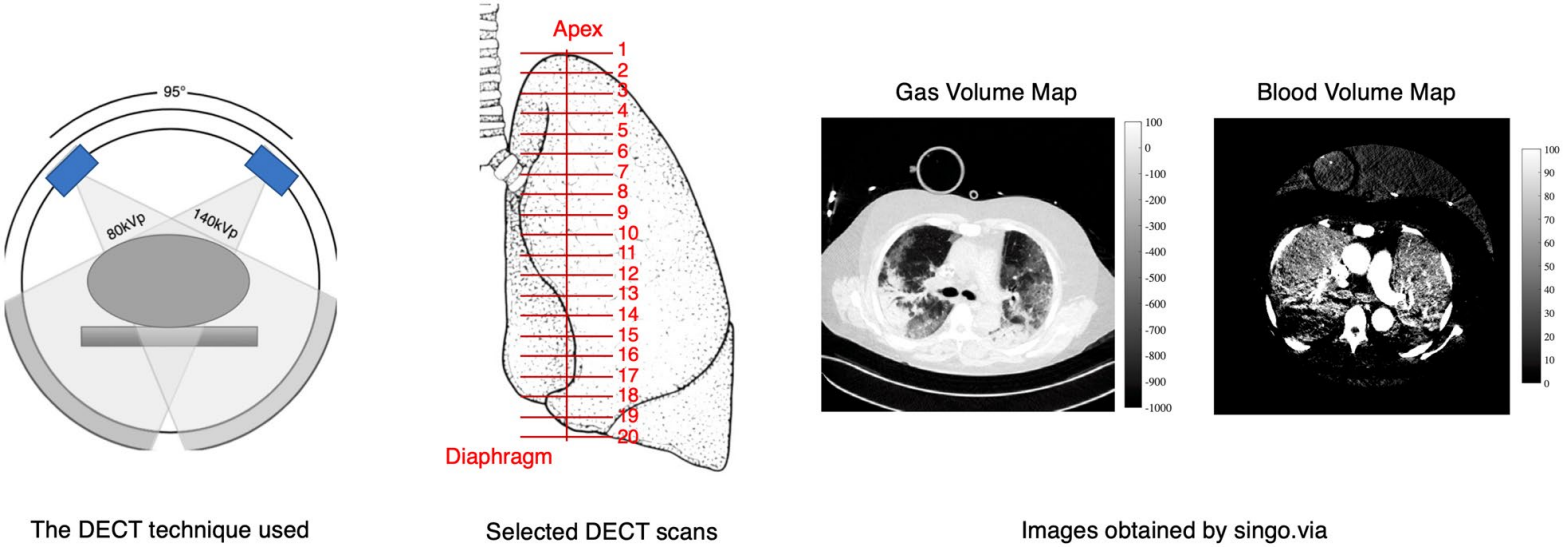
Dual-energy computed tomography (DECT) image acquisition and analysis. Left panel: DECT. A simplified representation of the DECT technique used, the SOMATOM Definition Flash, Siemens AG, Erlangen, Germany with two X-ray sources, characterized by two voltages (100 kVp and Sn 140 kVp), and two corresponding detectors rotating at a known constant angle. Central panel: Selected DECT scans. Coronal representation of a lung. Twenty DECT images, equally distributed between the apex and the diaphragmatic dome, were selected along the cranial–caudal axis. Both lungs were analyzed. Right panel: Images obtained by singo.via. Images used for further analysis. Representative example of pulmonary gas volume map and pulmonary blood volume map

We analyzed clinically indicated DECT performed on patients included in a prospective observational single-center study of critically ill COVID-19 patients admitted to the intensive care unit (ICU) at Uppsala University Hospital in Sweden between April 7, 2020, and January 18, 2021. The study was approved by the National Ethical Review Agency (EPM; No. 2020-01623) and performed according to the Declaration of Helsinki and its subsequent revisions. Patients older than 18 years with positive PCR test for SARS-CoV2 on nasal swab specimen and available chest DECT performed during the intensive care stay were analyzed. All patients were not vaccinated for SARS-CoV2. The scans were acquired on clinical indication in static lung conditions, covering the whole lung parenchyma in supine position (see Fig. 1). Comprehensive clinical data were collected at ICU admission and the day of DECT. A subgroup analysis was conducted, dividing the sampled population in two groups: the group of patients not treated with steroids (No-Steroids group) and the one treated with steroids (Steroids group). In June 2020, the early report of the RECOVERY dexamethasone trial [9, 10] was published and the treatment guidelines of the disease were changed accordingly. Consequently, all patients recruited after June 16, 2020, were treated with steroids, forming up the group labeled “Steroids.” Patients chronically treated with steroids for reasons different from COVID-19 but that received, during their ICU stay, a steroids dose comparable to or higher than Dexamethasone 6 mg/day were included in the Steroids group.

### DECT image analysis

**Fig. 2:**
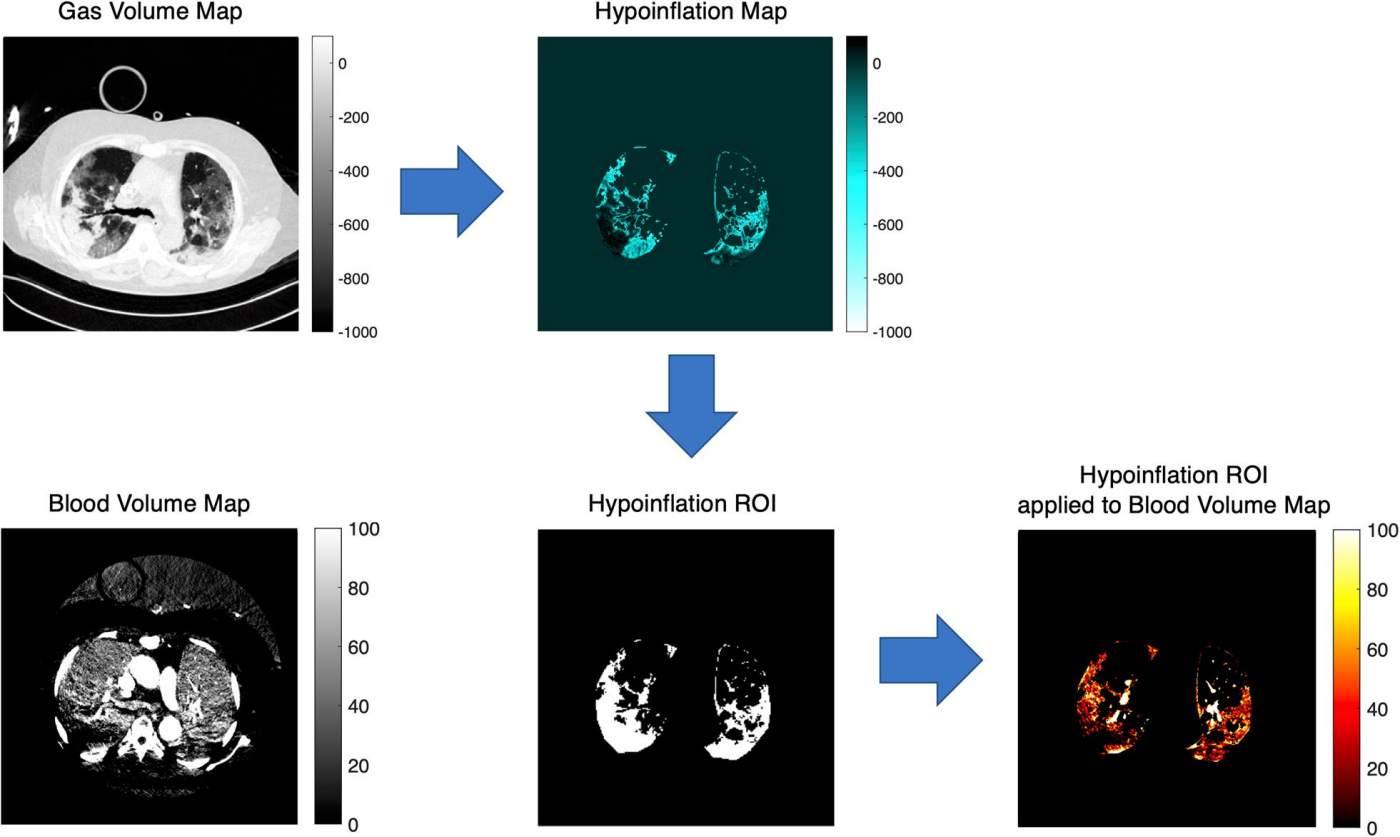
Dual-energy computed tomography image analysis. Pulmonary gas volume maps were characterized by a HU range of − 1000 to + 100 HU. Pulmonary blood volume maps were characterized by a HU range of − 100 to + 150 HU. To assess hypoxic pulmonary vasoconstriction, pulmonary blood volume distribution was assessed for the whole lung and for the hypoinflated lung, the latter characterized by poorly inflated and non-inflated lung regions (HU between − 500 and + 100). A region of interest for hypoinflated lung (Hypoinflation ROI) was obtained extrapolating hypoinflated areas (Hypoinflation Map) from the pulmonary gas volume map. Hence, the hypoinflated ROI was applied to pulmonary blood volume map to analyze blood volume distribution of the hypoinflated areas

**Fig. 3:**
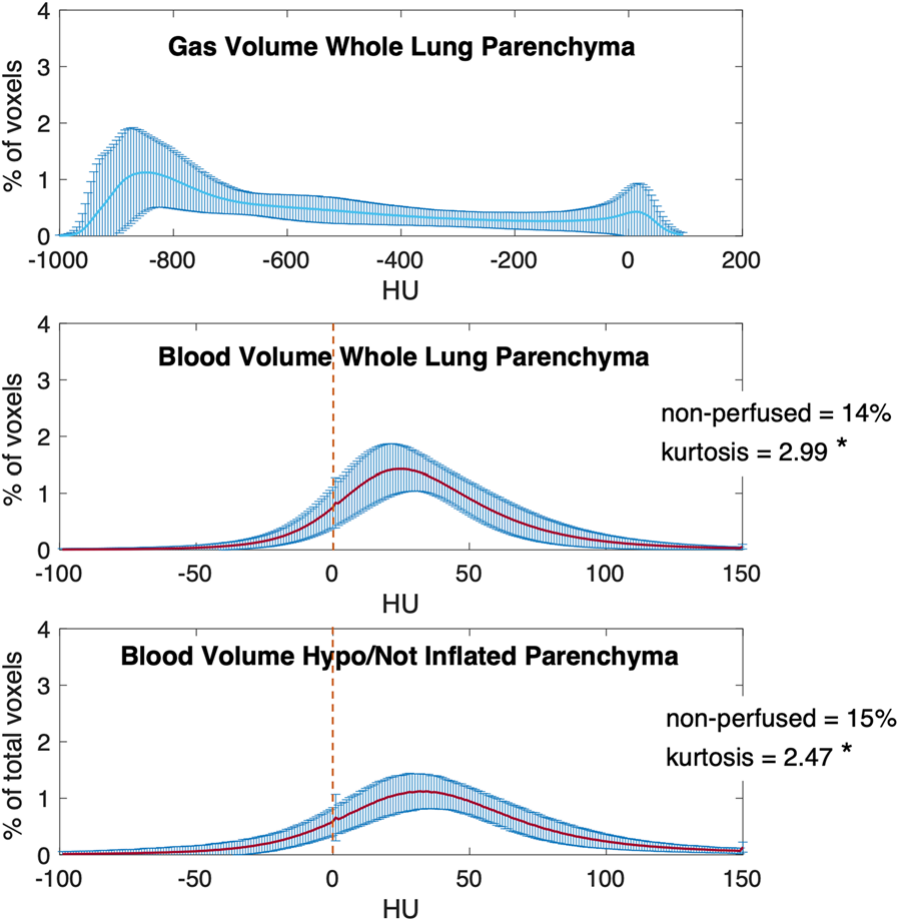
HU distribution for pulmonary gas volume map and blood volume maps, for the whole study population (*n* = 60 patients). Above: the HU distribution for pulmonary gas volume maps in the whole lung parenchyma; middle: HU distribution for pulmonary blood volume maps in the whole lung parenchyma; below: HU distribution for pulmonary blood volume maps in the hypoinflated lung parenchyma. *x*-axis: HU values. *y*-axis: percent of voxels compared to the total amount of voxels. Values represented as mean ± standard deviation (SD) or percent

Dual-energy computed tomography was always performed in supine position. For each raw DECT dataset, routine material-specific DECT maps were generated using a secondary workstation (Syngo.via Multimodality Workplace; Siemens Medical Solutions) that applied a three-material decomposition algorithm [18]. Two different series of images were selected and used for further analysis: the gas distribution (pulmonary gas volume) maps and the corresponding blood distribution (pulmonary blood volume) maps (see Fig. 1). Twenty images evenly spaced along the cranial–caudal axis and located between the apex and the diaphragmatic dome were analyzed (see Fig. 1). A manual segmentation of the lung parenchyma was applied, and big vessels, heart and mediastinal structures were excluded from the subsequent analysis. As regard to the pulmonary gas volume maps, the Hounsfield units (HU) range of − 1000 (gas) to + 100 (non-aerated lung) was considered, as done in previous studies (see Fig. 2). Four lung compartments were then defined [19]: hyperinflated (− 1000 to − 800 HU), normoinflated (− 800 to − 500 HU), poorly inflated (− 500 to − 100 HU) and non-inflated (− 100 to + 100 HU) lung. As regard to the pulmonary blood volume maps, we studied the range between − 100 and + 150 HU (see Fig. 2). Based on previous studies [20–22], zero HU was set as a limit between non-perfused (HU ≤ 0) and perfused lung compartment (HU > 0). Each compartment was separately computed and expressed as cm^2^ and as percentage of the total lung volume in each map [22]. From the bell-shaped curves characterizing the HU distribution of perfused blood volume maps, kurtosis was calculated as measure of both dispersion and morphology of the curves (see Fig. 3A) as done in previous studies [23–26]. Kurtosis belongs to the big chapter of the measures of variance, and it is commonly used to quantify both dispersion and morphology of density histograms derived from computed tomography images. A reduced kurtosis corresponds to a decreased homogeneity of pulmonary blood volume. The latter has been previously interpreted as indirect index of HPV efficiency [27]: a low homogeneity of pulmonary blood volume (low kurtosis) corresponding to a preserved HPV. To further assess the relation between and HPV, where a reduced pulmonary blood volume is expected in the hypoinflated lung, pulmonary blood volume was separately assessed for the whole lung and for the hypoinflated areas, the latter characterized by poorly inflated and non-inflated lung regions (i.e., HU ranging between − 500 and + 100 HU) (see Fig. 3).

### Lung weight analysis

Total lung volume (ml), total gas volume (ml) and lung weight (g) were computed [28–31]. The analysis was performed for the whole population as well as separately for the No-Steroids and the Steroids group.

### Statistical analysis

Continuous variables were reported as mean ± standard deviation (SD). The Wilcoxon signed-rank test (*α* = 0.05) was chosen as a nonparametric test for paired observations. The Wilcoxon rank-sum test (*α* = 0.05) was used as a nonparametric test in the case of independent observations. Fisher’s exact test (*α* = 0.05) was used to test statistically significant differences in the case of categorical variables. More details about Materials and methods are reported as Additional file 1: Data Supplement.

## Results

### Study population

Sixty patients affected by severe COVID-19 and in need of intensive care were analyzed in the study (see Table 1). The mean age was of 63 ± 9 years and 20% were women. At ICU admission, the ratio of partial pressure of arterial oxygen to inspired oxygen fraction at the day of the DECT was 127 ± 46 mmHg and the SAPS3 was 51 ± 9. During their ICU stay before DECT, 41% of patients were intubated and 22% experienced an acute thrombotic event. In this selected population, the 30-day mortality was 29%. Forty-three out of sixty patients (72%) received steroids as treatment of COVID-19-associated pneumonia (Dexamethasone 6 mg for 10 days) [9, 10]. In order to compare the severity of the disease in No-Steroid and Steroid patients, we analyzed a wide selection of clinical and biochemical markers (see Table 2). The Steroids group showed a lower incidence of thrombotic events: 14% compared to 41% (*p* = 0.03), a significantly lower alveolar–arterial difference of oxygen (AaDO_2_) the day of DECT compared to ICU admission (267 ± 99 mmHg vs 322 ± 106 mmHg, *p* = 0.04) as well as lower inflammation (e.g., IL-6 equal to 218 ± 155 pg/ml in the No-Steroids group vs 40 ± 19 pg/ml in the Steroids group, *p* = 0.01) compared to the No-Steroids group. The antithrombotic treatment (the dose of Dalteparin per kg) as well as the value of factor Xa (*p* = 0.11) did not differ between the two groups.

**Table 1:** Descriptive statistics for all patients included in the study (sample size = 60)

	Mean	SD
Age (years)	63	9
BMI	31	6

Female	*n*	%
	12	20

Clinical history before ICU stay	*n*	%
Previous pulmonary disease	16	27
Thrombotic events before ICU	6	10

At ICU admission	Mean	SD
Respiratory rate (bpm)	27	8
SAPS 3	51	9
Days of COVID-19 symptoms	11	3
F_I_O_2_	62	14
pH	7.45	0.06
PaCO_2_ [mm Hg]	35	5
PaO_2_/F_I_O_2_ ratio [mm Hg]	127	46
SpO_2_ [%]	94	2
AaDO_2_ [mm Hg]	325	105
Weight [kg]	97	18
Dalteparin [IE] /kg	144	42

	*n*	%
Intubated patients	21	35

On DECT day	Mean	SD
Days of invasive ventilation	7	7
PEEP [cm H_2_O]	11	4
Static compliance	46	20
Tidal volume [mL]/kg PBW	8	3
pH	7.42	0.08
PaCO_2_ [mm Hg]	42	1
PaO_2_/FIO_2_ ratio [mm Hg]	129	31
SpO_2_ [%]	94	3
AaDO_2_ [mm Hg]	272	97
Weight (kg)	96	18
Ferritin [µg/l]	2028	1398
D-dimer [ng/ml]	6	11
Interleukin-6 (IL-6) [pg/ml]	284	493
Factor Xa [KIE/l]	0.53	0.2

	*n*	%
Intubated patients	24	41
Spontaneously breathing patients	36	64
Acute thrombotic events before DECT	13	22

At ICU discharge	Mean	SD
Days of invasive ventilation	14	11
Days of ICU stay	14	11
Highest ferritin [µg/l]	2884	3054
Highest D-dimer [ng/ml]	13	26
Highest interleukin-6 (IL-6) [pg/ml]	228	380

	*n*	%
Alive 30 days after ICU	43	71
Invasive mechanical ventilation	32	53
Thrombotic events	16	27

Data were reported as mean ± standard deviation (SD) or numerosity (%)

Days of invasive ventilation, ventilatory settings and respiratory mechanics values refer only to the subgroup of mechanically ventilated patients. *Spontaneously breathing patients* refers to patients with preserved respiratory drive both with and without invasive mechanical ventilation

BMI, body mass index; SAPS 3, the simplified acute physiology score 3; F_I_O_2_, inspiratory fraction of oxygen; PaO_2_, partial pressure of arterial oxygen; SpO_2_, peripheral oxygen saturation; PCO_2_, partial pressure of arterial carbon dioxide; AaDO_2_, alveolar arterial difference of oxygen

**Table 2:** Descriptive statistics for the two patients’ subgroups: No-Steroids group (left, simple size = 17) and Steroids group (right, simple size = 43)

	No-Steroids (*n* = 17)		Steroids (*n* = 43)		
	Mean	SD	Mean	SD	Wilcoxon rank-sum test (*α* = 0.05)
Age (years)	62	9	64	9	0.37
BMI	31	6	31	6	1

	*n*	%	*n*	%	Fisher’s exact test (*α* = 0.05)
Female	1	6	11	26	0.15

Clinical history before ICU stay	*n*	%	*n*	%	
Previous pulmonary disease	5	29	11	26	0.76
Thrombotic events before ICU	2	7	4	9	1

At ICU admission	Mean	SD	Mean	SD	Wilcoxon rank-sum test (*α* = 0.05)
Respiratory rate (bpm)	28	9	28	7	0.72
SAPS 3	51	7	51	11	0.97
Days of COVID-19 symptoms	11	3	11	3	0.84
F_I_O_2_	60	13	63	16	0.54
pH	7.46	0.05	7.45	0.06	0.56
PaCO_2_ [mm Hg]	36	5	35	6	0.22
PaO_2_/F_I_O_2_ ratio [mm Hg]	138	65	124	36	0.88
SpO_2_ [%]	93	3	94	2	0.42
AaDO_2_ [mm Hg]	326	102	332	106	0.9
Weight [kg]	100	20	96	17	0.49
Dalteparin [IE] /kg	133	45	148	41	0.11

	*n*	%	*n*	%	Fisher’s exact test (*α* = 0.05)
Intubated patients	5	29	16	37	0.77

On DECT day	Mean	SD	Mean	SD	Wilcoxon rank-sum test (*α* = 0.05)
Days from start of symptoms	16	8	13	6	0.45
Days from steroids therapy start	0	0	4	3	< 0.01
Days of invasive ventilation	9	6	6	7	0.31
PEEP [cm H_2_O]	12	4	10	4	0.08
Static compliance	43	24	48	18	0.49
Tidal volume [mL]/kg PBW	8	2	9	4	0.37
pH	7.43	0.08	7.41	0.08	0.45
PaCO_2_ [mm Hg]	42	12	42	10	0.95
PaO_2_/FIO_2_ ratio [mm Hg]	123	25	132	34	0.43
SpO_2_ [%]	93	3	94	2	0.86
AaDO_2_ [mm Hg]	284	81	267	99	0.63
Weight (kg)	100	21	95	17	0.43
Ferritin [µg/l]	2315	1676	1694	950	0.27
D-dimer [ng/ml]	9	13	5	11	0.72
Interleukin-6 (IL-6) [pg/ml]	218	155	40	19	0.21
Factor Xa [KIE/l]	0.55	0.23	0.53	0.2	0.18

	*n*	%	*n*	%	Fisher’s exact test (*α* = 0.05)
Intubated patients	8	47	16	37	0.77
Spontaneously breathing patients	9	52	27	69	1
Acute thrombotic events before DECT	7	41	6	14	0.03*

At ICU discharge	Mean	SD	Mean	SD	Wilcoxon rank-sum test (*α* = 0.05)
Days of invasive ventilation	12	8	5	3	0.14
Days of ICU stay	16	6	8	4	0.45
Highest ferritin [µg/l]	5067	4257	2046	1974	0.03*
Highest D-dimer [ng/ml]	26	42	10	20	0.9
Highest interleukin-6 (IL-6) [pg/ml]	449	564	95	71	0.01*

	*n*	%	*n*	%	Fisher’s exact test (*α* = 0.05)
Alive 30 days after ICU	12	71	31	72	0.49
Invasive mechanical ventilation	10	59	22	51	0.77
Thrombotic events	8	47	8	19	0.04*

Data were reported as mean ± standard deviation (SD) or numerosity (%). The analyzed variables are the same as in Table 1. Wilcoxon rank-sum test to detect statistical differences for continuous variables; (*α* = 0.05); Fisher’s exact test to detect statistical differences for categorical variables; (*α* = 0.05); *: to mark statistically significant differences.

### DECT image analysis

**Fig. 4:**
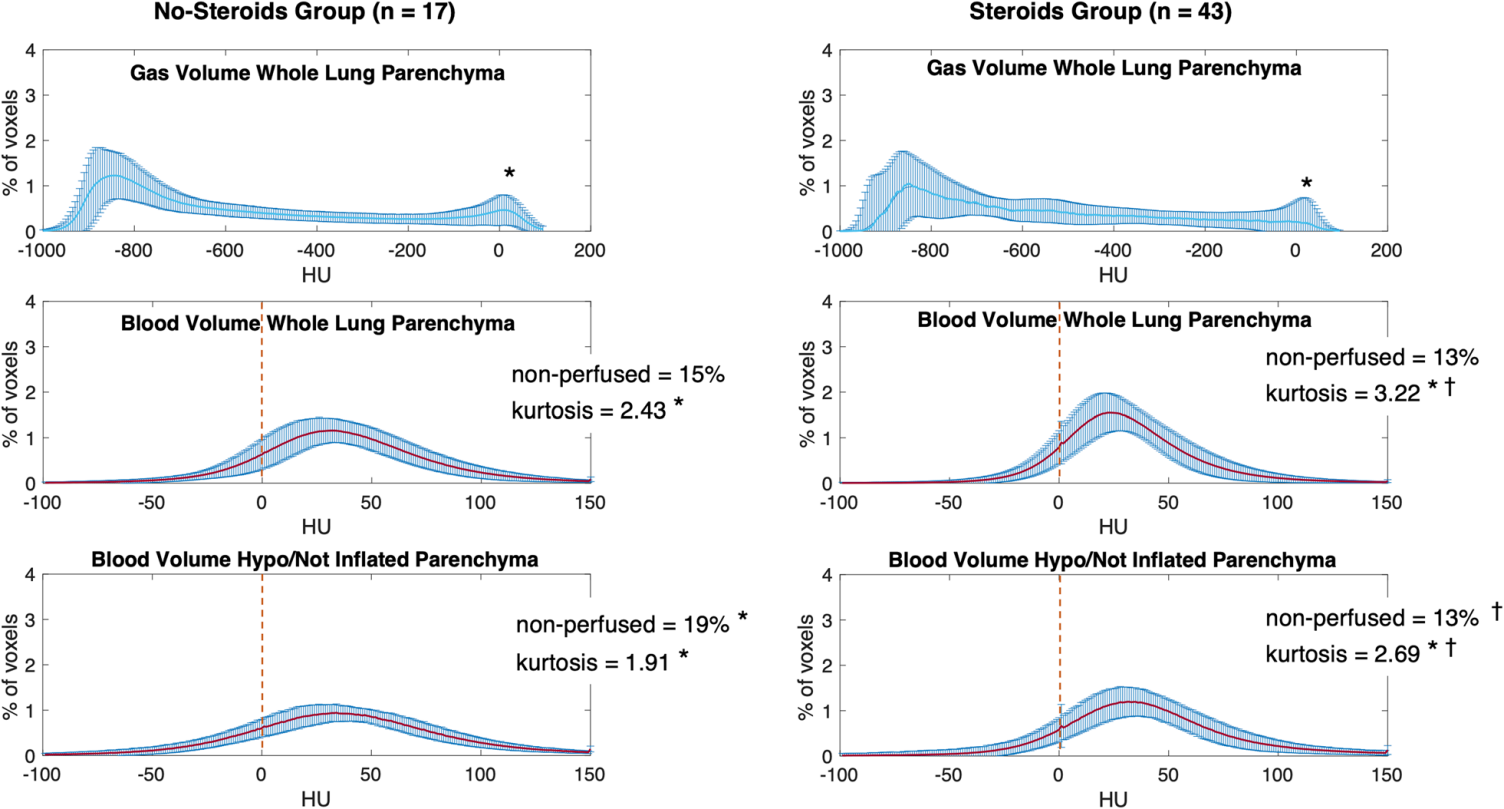
HU distribution for pulmonary gas volume map and blood volume maps, separately for the No-Steroids group (*n* = 17 patients; Panel **A**) and the Steroids group (*n* = 43 patients; Panel **B**). Above: the HU distribution for pulmonary gas volume maps in the whole lung parenchyma; middle: HU distribution for pulmonary blood volume maps in the whole lung parenchyma; below: HU distribution for pulmonary blood volume maps in the hypoinflated lung parenchyma. *x*-axis: HU values. *y*-axis: percent of voxels compared to the total amount of voxels. Values represented as mean ± standard deviation (SD) or percent. *: for statistical differences between whole lung parenchyma (middle) and hypoinflated parenchyma (Below); †: for statistical differences between the No-Steroids group (Panel A) and the Steroids group (Panel B)

The HU analysis describing the gas distribution in the whole lung parenchyma showed two peaks corresponding to the hyperinflated and to the non-inflated HU compartments (see Fig. 3 and Additional file 2: Table E1). The analysis of pulmonary blood volume distribution showed 12% of non-perfused areas in the whole lung and 11% in the hypoinflated lung (*p* = 0.06). The HU distribution for pulmonary blood volume maps had a significantly lower kurtosis (lower homogeneity) in the hypoinflated lung compared to the whole lung parenchyma. This was true for the whole population (2.47 vs 2.99) as well as for the two analyzed subgroups: No-Steroids (1.91 vs 2.43) and Steroids group (2.69 vs 3.22) (see Figs. 3, 4, Additional file 2: Figure E1 and Tables E1–E2). Patients treated with steroids showed a smaller amount of non-inflated lung, compared to patients not exposed to steroids (10% vs 13%, *p* = 0.01). However, steroids did not affect the volume of hyperinflated lung (26% vs 27% of total lung volume, *p* = 0.24). Hypoinflated areas of the lung showed a significantly lower non-perfused lung parenchyma in the Steroids group compared to the No-Steroids group (13% vs 19% of total lung volume, *p* < 0.01). Exposure to steroids was associated with an increased kurtosis (increased homogeneity) of the HU distribution of pulmonary blood volume within the lung parenchyma, regardless of the degree of inflation (2.43 vs 3.22, *p* < 0.01 for the whole lung and 1.91 vs 2.69, *p* < 0.01 for the hypoinflated lung). The high kurtosis (high homogeneity of pulmonary blood volume distribution) found in the Steroids groups might suggest a jeopardized HPV [27] (see Figs. 4, Additional file 2: Figure E1 and Table E2).

### Lung weight analysis

The analysis of total lung volume, gas volume and lung weight is reported in Tables 3, 4, 5 and Additional file 2: Figure E2. The whole population showed a mean lung weight of 966 ± 303 g and a mean total gas volume of 1436 ± 718 ml. The total lung volume was 2248 ± 894 ml of which 30% was hyperinflated and 10% was non-inflated. By dividing the patients based on their exposure to steroids, significant differences were observed for lung volume and lung weight. The group of patients exposed to steroids showed a significantly lower lung weight (913 g vs 1123 g, *p* = 0.01) as well as a significantly lower volume of non-inflated lung parenchyma compared to patients not exposed to steroids (9% vs 11% of the total lung volume, *p* = 0.02). The total gas volume inside the lung was not affected by steroids (1425 vs 1465 ml, *p* = 0.71) (Table 4).

**Table 3:** Lung volume (ml) calculated for the whole lung parenchyma as well as for each HU compartment (hyper-, normally, poorly and non-inflated)

	Lung volume (ml)		Lung volume (%)	
	Mean	SD	Mean	SD
*Panel A*				
All patients				
Total lung	2248	894		
Hyperinflated	676	614	30	18
Normally inflated	822	409	36	11
Poorly inflated	535	249	24	11
Non-inflated	216	200	10	10

	Lung volume (ml)		Lung volume (%)	
	Mean	SD	Mean	SD
*Panel B*				
No-Steroids				
Total lung	2377	974		
Hyperinflated	679	468	29	13
Normally inflated	862	400	36	9
Poorly inflated	567	262	24	8
Non-inflated	269	153	11	8

	Lung volume (ml)			Lung volume (%)		
	Mean	SD	*α* = 0.05	Mean	SD	*α* = 0.05
*Panel C*						
Steroids						
Total lung	2198	868	0.59			
Hyperinflated	674	668	0.44	31	19	0.59
Normally inflated	806	416	0.60	36	11	0.78
Poorly inflated	522	245	0.67	24	12	0.45
Non-inflated	196	213	0.01	9	11	0.02

Panel A: whole study population (*n* = 60); Panel B: No-Steroids group (*n* = 17); Panel C: Steroids group (*n* = 43). Each HU compartment was reported as absolute values as well as percentage of the total lung parenchyma. Data were reported as mean ± standard deviation (SD). Wilcoxon rank-sum test was used to detect statistical differences (*α* = 0.05); *: to mark statistically significant differences.

**Table 4:** Gas volume (ml) calculated for the whole lung parenchyma as well as for each HU compartment (hyper-, normally, poorly and non-inflated)

	Gas volume (ml)		Gas volume (%)	
	Mean	SD	Mean	SD
*Panel A*				
All patients				
Total lung	1436	718		
Hyperinflated	617	557	43	18
Normally inflated	580	281	40	13
Poorly inflated	203	93	14	9
Non-inflated	36	13	3	1

	Gas volume (ml)		Gas volume (%)	
	Mean	SD	Mean	SD
*Panel B*				
No-steroids				
Total lung	1465	671		
Hyperinflated	614	421	42	14
Normally inflated	609	275	42	11
Poorly inflated	207	99	14	7
Non-inflated	36	14	2	1

	Gas volume (ml)			Gas volume (%)		
	Mean	SD	*α* = 0.05	Mean	SD	*α* = 0.05
*Panel C*						
Steroids						
Total lung	1425	743	0.71			
Hyperinflated	619	607	0.48	43	20	0.49
Normally inflated	568	286	0.62	40	14	0.66
Poorly inflated	201	91	0.88	14	10	0.39
Non-inflated	36	12	0.74	3	1	0.37

Panel A: whole study population (*n* = 60); Panel B: No-Steroids group (*n* = 17); Panel C: Steroids group (*n* = 43). Each HU compartment was reported as absolute values as well as percentage of the total lung parenchyma. Data were reported as mean ± standard deviation (SD). Wilcoxon rank-sum test was used to detect statistical differences (*α* = 0.05); *: to mark statistically significant differences

## Discussion

To date, despite established evidence of a reduced mortality in severe COVID-19 patients treated with steroids compared to a control population [9, 10], no studies investigated the effects of steroids on the lungs in this patients’ population. In the current study, for the first time, we introduced the use of dual-energy computed tomography to study the effects of steroids on lung ventilation and perfusion as well as on lung weight in patients affected by severe COVID-19 and in need of intensive care. The main findings of the current study can be summarized as follows: (1) the use of steroids is associated with a high kurtosis of lung perfusion in the hypoventilated regions of the lung. This suggests that steroids might counteract hypoxic pulmonary vasoconstriction; (2) steroids reduced lung weight, possibly originated by a decreased edema; (3) steroids improve regional gas distribution; (4) despite potential effects on hypoxic pulmonary vasoconstriction, gas change was improved as evidenced by a reduction in AaDO2, suggesting that the effects of steroids to reduced edema and improve gas distribution predominated.

In clinical contexts different from COVID-19, as high-altitude pulmonary edema (HAPE), steroids have been demonstrated to reduce pulmonary arterial systolic pressure, presumably counteracting hypoxic pulmonary vasoconstriction [12, 13]. At the same time, steroids reduce inflammation and lung edema [14]. The possible known mechanisms of action of steroids on lungs pathophysiology are multifold. Steroid therapy is known for improving pulmonary function in preterm newborns by augmenting nitric oxide-mediated vessels relaxation as well as by stimulating surfactant production [32]. Steroids prevent capillary leak, stimulating alveolar sodium and water reabsorption [14], and enhance nitric oxide availability in the lung parenchyma [33]. There is a still ongoing debate about the possible benefits of their anti-inflammatory and antifibrotic properties on patients with acute respiratory distress syndrome [34, 35]. The current study is the first investigating the possible effects of steroids on lungs in critically ill patients affected by severe COVID-19.

Interestingly, in the current study, the Steroids and the No-Steroids groups were similar to each other in all respects except for the indexes of inflammation and the onset of thromboembolic events during their ICU stay (see Table 2). Patients treated with steroids showed significantly lower inflammatory markers and, at the same time, a lower occurrence of thromboembolic events (19% vs 47%, *p* = 0.04). The dose of antithrombotic drugs as well as the levels of factor Xa and D-dimers could not justify such differences. Although concomitant causes, potentially influencing the prothrombotic state of these patients, cannot be ruled out, the immunomodulatory effects and antithrombotic properties of steroids [36] can be a possible explanation for these differences [37]. Although not reaching statistical significance, patients treated with steroids showed an improved oxygenation (PaO_2_/F_I_O_2_: 132 vs 123, *p* = 0.43), a reduction in alveolar arterial difference of oxygen (AaDO_2_ 326 mmHg vs 332 mmHg, *p* = 0.9) and ameliorate static compliance (48 vs 43, *p* = 0.49) as well as less days of mechanical ventilation and days of ICU stay. A significant reduction in AaDO_2_ followed the treatment with steroids (322 ± 106 mmHg at admission vs 267 ± 99 mmHg at DECT, *p* = 0.04). The sample was underpowered to investigate a difference in mortality between the two groups.

### Effects of steroids on hypoxic pulmonary vasoconstriction

**Fig. 5:**
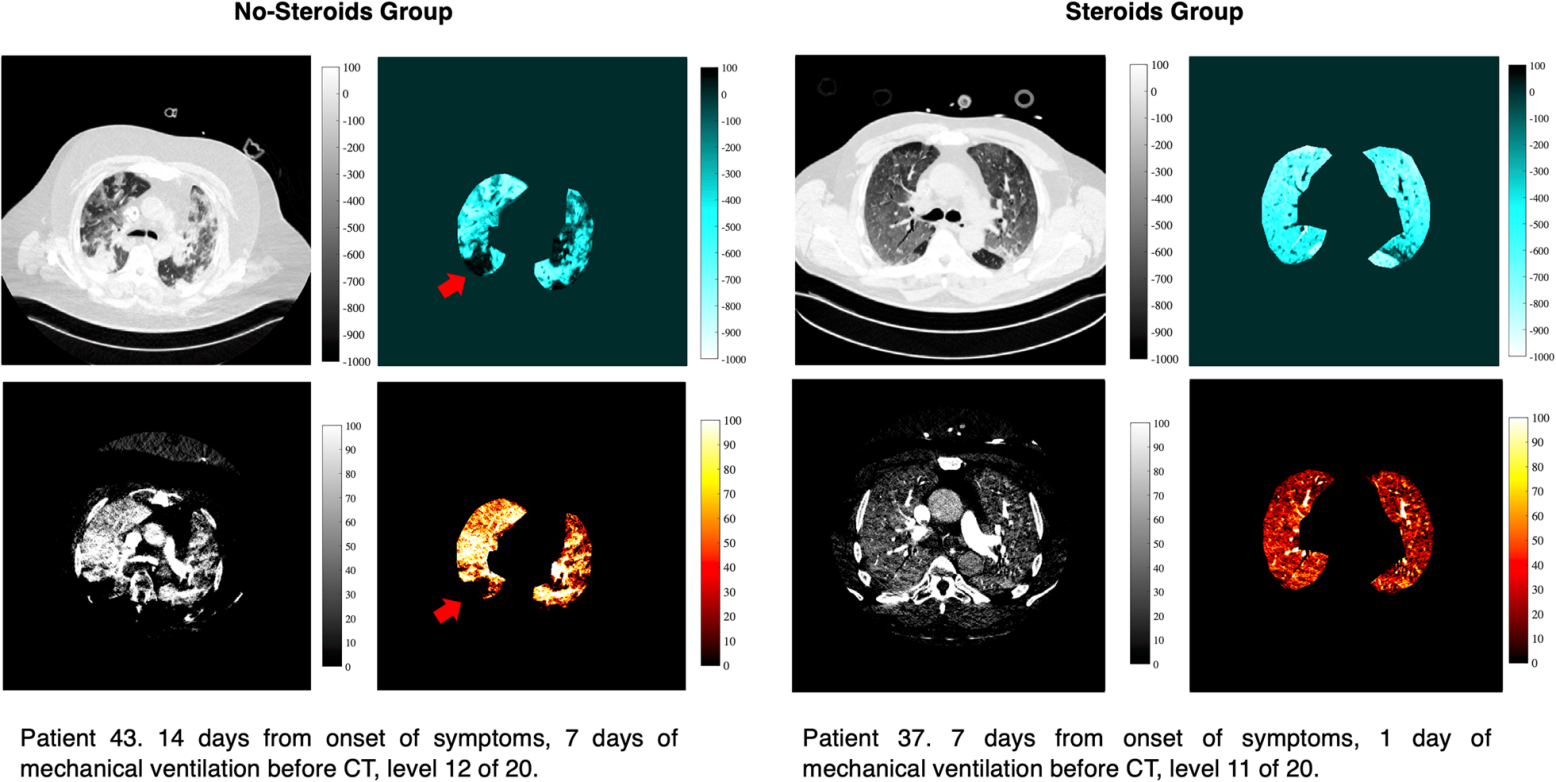
Representative example of DECT scans collected on the No-Steroids group (left) and the Steroids group (right). On the left: Patient not treated with steroids (No-Steroids group). On the right: Patient treated with steroids (Steroids group). For both No-Steroids and Steroids group, upper images show a pulmonary gas volume image (left) and the corresponding map (right); lower images show a pulmonary blood volume image (left) and the corresponding map (right). Different colormaps have been used to emphasize different degree of pulmonary gas and blood volume. For pulmonary gas volume maps, the range of HU is between − 1000 and + 100 HU. For pulmonary blood volume maps, the range of HU is between 0 and + 100 HU. 0 HU corresponding to non-perfused areas, 100 HU corresponding to highly perfused areas. The red arrow indicates the areas of preserved hypoxic pulmonary vasoconstriction where the areas of low pulmonary blood volume correspond to the areas of low inflation

HPV is a homeostatic mechanism, peculiar of the pulmonary vasculature. Intrapulmonary arterioles constrict in response to alveolar hypoxemia, diverting blood from injured lung toward better ventilated lung regions. Thereby, HPV optimizes ventilation/perfusion match and improves oxygen delivery [38, 39]. A dysregulated HPV has been hypothesized in ARDS [40] and COVID-19 patients [15, 41]. The current study was neither designed to directly investigate the mechanisms of action of steroids on HPV in COVID-19 patients nor planned to grade the HPV derangement induced by COVID-19. However, our data suggest that in patients affected by severe COVID-19 the use of steroids might jeopardize HPV (see Figs. 4, 5 and Additional file 2: Table E2). A damped HPV is apparently not in line with the known beneficial effects of steroids observed in severe COVID-19 patients [9, 42]. However, this can mitigate the risk for right ventricular failure, one of the major determinants of poor clinical outcomes in ARDS [43, 44]. Moreover, other concomitant beneficial mechanisms induced by steroids as anti-inflammatory and immunomodulatory effects, as well as the reduction in lung edema, demonstrated in this study, might also compensate for a worsened HPV and an exacerbated ventilation/perfusion mismatch. Although HPV is the primary active mechanism influencing regional lung perfusion, other concomitant factors can have a role. Among others, superinfections by Gram-negative bacteria, potentially less hindered in patients treated with steroids, could possibly attenuate HPV [45]. At the same time, direct mechanical effects as well as undefined intracellular signaling can be listed among non-hypoxia-related mechanisms affecting regional lung perfusion [46, 47].

### Effects of steroids on lung edema

At the pulmonary level, steroids prevent inflammatory capillary leak and edema, stimulating alveolar sodium and water reabsorption [14]. We demonstrated a reduced lung weight as well as lower volume of non-inflated lung in patients treated with steroids compared to control (see Table 3, 5 and Additional file 2: Figure E2).

**Table 5:** Lung weight (g) calculated for the whole lung parenchyma as well as for each HU compartment (hyper-, normally, poorly and non-inflated)

	Weight (g)		Weight (%)	
	Mean	SD	Mean	SD
*Panel A*				
All patients				
Total lung	966	303		
Hyperinflated	114	76	12	10
Normally inflated	301	148	31	11
Poorly inflated	353	170	37	10
Non-inflated	198	150	20	13

	Weight (g)		Weight (%)	
	Mean	SD	Mean	SD
*Panel B*				
No-Steroids				
Total lung	1123	328		
Hyperinflated	122	66	11	7
Normally inflated	310	145	27	9
Poorly inflated	417	184	37	7
Non-inflated	291	151	25	11

	Weight (g)			Weight (%)		
	Mean	SD	*α* = 0.05	Mean	SD	*α* = 0.05
*Panel C*						
Steroids						
Total lung	913	276	0.01*			
Hyperinflated	108	80	0.57	12	11	0.93
Normally inflated	290	151	0.85	32	12	0.34
Poorly inflated	323	165	0.60	35	11	0.44
Non-inflated	192	152	0.01*	21	14	0.03

Panel A: whole study population (*n* = 60); Panel B: No-Steroids group (*n* = 17); Panel C: Steroids group (*n* = 43). Each HU compartment was reported as absolute values as well as percentage of the total lung parenchyma. Data were reported as mean ± standard deviation (SD). Wilcoxon rank-sum test was used to detect statistical differences (*α* = 0.05); *: to mark statistically significant differences

Although not affecting global gas volume (see Tables 3, 5), steroids improved the homogeneity of gas distribution within the lung parenchyma (see Fig. 4 and Additional file 2: Table E2). The exposure to steroids reduced the non-inflated, atelectatic lung compartment. This is reflected in a significantly reduced AaDO_2_ following treatment with steroids (322 ± 106 mmHg at admission vs 267 ± 99 mmHg at DECT, *p* = 0.04). These findings can also be interpreted as a consequence of a reduced lung edema and inflammation following treatment with steroids. Thus, although there was evidence for reduced HPV the beneficial effects of steroids acting to reduce edema and improve gas distribution on gas exchange predominated.

Alongside emerging understanding of the pulmonary vascular involvement in COVID-19 patients, the key mechanism of the most immediately dangerous consequence of COVID-19 (hypoxemia) has been ascribed to the concurrent disruption of the tri-compartmental model of lung oxygenation [48]. Consequently, research has progressively focused upon the ventilation/perfusion mismatch. Mauri et al. [49], in a small cohort of COVID-19 patients studied using electrical impedance tomography, reported that ventilation/perfusion mismatch was elevated and mainly due to non-perfused but ventilated units. Later, using a computational model, Busana et al. [50] attributed hypoxemia of COVID-19 to a marked hyperperfusion of poorly ventilated lung regions.

Given its availability in clinical practice, chest contrast-enhanced DECT is emerging as a promising tool for studying lung physiology [16, 17]. The strength of DECT is that it simultaneously provides information about both gas and blood distribution and, consequently, has great potential in describing ventilation/perfusion dysregulation in COVID-19 [41]. Recently, Ball et al. [20] performed the first quantitative study regarding the distribution of gas volume and blood in the lungs of patients affected by COVID-19 by using DECT, reporting several deviations from normal physiology, among which the blood volume distribution in non-aerated tissue mass.

This is the first study based on chest DECT investigating the functional effects of steroids on lungs in patients affected by severe COVID-19. Compared to the previous literature, the current study took a step forward, not only investigating the regional distribution of pulmonary gas volume and blood volume in COVID-19, but also characterizing the effects that steroids have on them. Our results suggested that a deranged HPV or a reduced lung edema might modify the ventilation/perfusion mismatch characterizing COVID-19 lungs.

Limitations of the study can be listed as follows. Being an observational study, the analysis is based on DECT performed on the basis of a clinical indication and this might imply a selection bias. However, all COVID-19 patients admitted to our ICU, with very few exceptions, underwent either computed tomography with contrast or DECT. All were performed in supine position. As reported in Table 2, the time between the symptoms start and DECT was not different between the two groups (16 ± 8 days for the No-Steroids group vs 13 ± 6 days for the Steroids group, *p* = 0.45). Moreover, in the Steroids group, the DECT examinations included in the study were all performed within 4 ± 3 days from the start of the steroid treatment (see Table 2). With few exceptions with clear contraindications, pronation was routinely applied for all patients, already before ICU stay. Therefore, the exact time spent in pronation by each patient cannot be reliably quantified. The execution of the DECT was performed not before one hour from the end of the eventual preceding pronation phase due to the necessity of observing whether the patient was sufficiently stable for the transport to the CT facility. All patients in the No-Steroids group were admitted before June 16, 2020; after that date, the COVID-19 patients admitted to the ICU were treated with steroids (Steroids group) as a consequence of a change of treatment guidelines. The fact that steroids have been introduced at a specific time of the pandemic might introduce a chronology bias into the study. As a matter of fact, the SARS-CoV2 pandemic during its course has been characterized by a continuous change of treatment strategies and clinical routines that affect the generalizability of many studies on COVID-19. However, in our study, all the recruited patients were not vaccinated for SARS-CoV2, they were all affected by the same SARS-CoV2 variant (the original Wuhan variant) and, in the studied time lapse, there were not concomitant antiviral/anti-inflammatory treatments specific to COVID-19 other than steroids. Given the demonstrated positive effects of steroids and their routine use in severe COVID-19 patients [9], a prospective study comparing patients exposed or not exposed to steroids would have entailed unsolvable ethical issues. Given the design of the study, the direct causality between steroids and a deranged HPV could not be directly investigated. We did not directly study how steroids influence the pathophysiological mechanisms of HPV. Moreover, a deranged HPV is not the only factor interfering with the regional distribution of perfused blood volume, e.g., pulmonary thrombotic events, lung heterogeneity or interstitial edema are concomitant causes also affecting blood distribution and shunt. However, of all factors potentially aggravating shunt, HPV is the only one influencing, in a targeted manner, hypo-/non-inflated lung regions. The DECT scanner does not reach the resolution necessary to observe the behavior of single capillaries. For this reason, the attenuation value of one voxel portrayed the behavior of many alveoli and of the complex mesh of capillary vessels surrounding them. In this respect, the choice of focusing the analysis to the subpopulation of voxels subtending hypo- or non-inflated areas reduces the possible error deriving from the mechanical interaction between the inflated alveoli and the neighboring capillary units, being them in a similar inflation status. Although intriguing and supported by indirect evidences (i.e., lung imaging), the hypothesized parallelism between the mechanisms of action of steroids in HAPE and COVID-19 needs further investigations [51]. Steroids have pleiotropic effects [11] and the current study investigated only some possible consequences of pharmacodynamic mechanisms characterizing steroids. Chest DECT provides static information about gas volume at one predefined moment. Pulmonary gas volume maps are widely used in the literature to infer ventilation distribution [19]. The same applies to DECT pulmonary blood volume maps [52]. DECT generates images describing blood volume distribution within the lung at one single time point: For this reason, these images should only be indirectly and cautiously used to infer lung perfusion. Despite its limitations, the present study is the first one shedding light on the effects of steroids on the regional distribution of pulmonary gas volume and blood volume in COVID-19 patients.

## Conclusions

Chest DECT can provide unique functional information and visualize the effects of pharmacodynamic mechanisms of drugs on lung parenchyma. Taking advantage of chest DECT, we show changes in pulmonary gas volume and blood volume that might stand for an association between the use of steroids and hypoxic pulmonary vasoconstriction in severe COVID-19 patients in need of intensive care. Steroids can influence ventilation/perfusion mismatch. Moreover, treatment with steroids is associated with a reduced lung edema and consequently improves regional distribution of gas volume within the lungs of severe COVID-19 patients.

## Supplementary Information

**Additional file 1**. Supplementary Material and Methods.

**Additional file 2. Table E1**. Descriptive statistics for HU distribution for pulmonary gas volume and blood volume maps. Whole population (n = 60). Comparison between the whole parenchyma and hypoinflated areas. Data were reported as mean ± standard deviation (SD) as well as percent. Wilcoxon rank-sum test was used to test statistical differences between the whole parenchyma and the hypoinflated one (α = 0.05); *: to mark statistically significant differences. **Table E2**. Descriptive statistics for HU distribution for pulmonary gas volume and blood volume maps. Separately for the No-Steroids group (n = 17, Panel A) and the Steroids group (*n* = 43, Panel A) Comparison between the whole parenchyma and hypoinflated areas (Panel A and B) as well as between No-Steroids and Steroids group (Panel C). Data were reported as mean ± standard deviation (SD) as well as percent. Wilcoxon rank-sum test was used to test statistical differences (*α* = 0.05); *: to mark statistically significant differences. **Figure E1**. Not Perfused Area % vs Kurtosis scatter plot in the No-Steroids (left) and the Steroids (right) group. Data reported for all the included patients, divided in No-Steroids (left) and Steroids (right), in the whole parenchyma (blue) and the hypo-/non-inflated lung (red). The stars represent the mean values for each of the four groups of data. The group treated with steroids (right /stars) has significantly higher values of kurtosis than patients in the No-Steroids group (left/stars). The Not Perfused parenchyma in the hypo/not inflated lung is larger in the No-Steroids group (left red) compared to the Steroids group (right red). No differences are detectable in the Whole lung parenchyma (blue) between Steroids and No-Steroids group. These findings can be interpreted as preserved hypoxic pulmonary vasoconstriction (HPV) in the No-Steroids group and jeopardized HPV in the Steroids group. **Figure E2**. Distribution of total lung weight in the two analyzed groups: *N*-Steroids (left) and Steroids (right). Each orange circle indicates the estimated lung weight in grams for each included patient. The mean values are indicated as black lines and reported as values.

## Data Availability

Data are available from the corresponding author on reasonable request after appropriate ethical and data safety requirements are met (https://doi.org/10.17044/scilifelab.14229410).

## Abbreviations

BMI: Body mass index
COVID-19: Coronavirus disease 2019
CT: Computed tomography
DECT: Dual-energy computed tomography
F_I_O_2_: Inspiratory fraction of oxygen
HAPE: High-altitude pulmonary edema
HU: Hounsfield units
HPV: Hypoxic pulmonary vasoconstriction
ICU: Intensive care unit
IL-6: Interleukin 6
IQR: Interquartile range
PCO_2_: Partial pressure of arterial carbon dioxide
PaO_2_: Partial pressure of arterial oxygen
PaO_2_/F_I_O_2_: Ratio of arterial oxygen partial pressure to fractional inspired oxygen
PCR: Polymerase chain reaction
SAPS 3: Simplified acute physiology score 3
SpO_2_: Peripheral oxygen saturation
