# Supplementary material for: Chest dual-energy CT to assess the effects of steroids on lung function in severe COVID-19 patients
Source: Crit Care. 2022 Oct 25;26:328. doi: 10.1186/s13054-022-04200-z (PMC9595078; doi:10.1186/s13054-022-04200-z)
Supplement: Supplementary file 1 — Additional file 1. Supplementary Material and Methods. [file 13054_2022_4200_MOESM1_ESM.docx]

**Chest dual energy CT to assess the effects of steroids on lung function in severe COVID-19 patients – Supplementary material**

Gaetano Perchiazzi MD, PhD ^1,2,3^ ; Aleksandra Larina, MD ^1,3^ ; Tomas Hansen, MD, PhD ^4^; Robert Frithiof MD, PhD ^1,3^ ; Michael Hultström MD, PhD ^1,3,5^ ; Miklos Lipcsey, MD, PhD ^1,2,3^ ; Mariangela Pellegrini, MD, PhD ^1,2,3^.

^1^ Anesthesiology and Intensive Care Medicine, Department of Surgical Sciences, Uppsala University, Sweden;

^2^ Hedenstierna Laboratory, Department of Surgical Sciences, Uppsala University, Sweden;

^3^ Department of Anesthesia, Operation and Intensive Care, Uppsala University Hospital, Uppsala, Sweden

^4^ Section of Radiology, Department of Surgical Sciences, Uppsala University, Uppsala, Sweden;

^5^ Integrative Physiology, Department of Medical Cell Biology, Uppsala University, Uppsala, Sweden.

**MATERIALS AND METHODS**

**Study population**

We analyzed clinically indicated DECT performed on patients included in a prospective observational single center study of critically ill COVID-19 patients admitted to the intensive care unit (ICU) at Uppsala University Hospital in Sweden between April 7^th^, 2020 and January 18^th^, 2021. The study was approved by the National Ethical Review Agency (EPM; No. 2020-01623) and performed according to the Declaration of Helsinki and its subsequent revisions. STROBE guidelines were followed for data reporting. Informed consent was obtained either directly from the patient or, if the patient was unable to give consent, from patient’s next of kin. Patients older than 18 years with positive PCR test for SARS-CoV2 on nasal swab specimen and available chest DECT performed during the intensive care stay were analyzed. Data gathered from patients that underwent chest dual energy CT (chest DECT, SOMATOM Definition Flash, Siemens AG, Erlangen, Germany) were selected for the current study.

**Dual Energy CT scans**

Dual energy computed tomography was always performed in supine position. Chest DECT scans were performed when clinically indicated and feasible, hence at different stages of the disease. The DECT scans were performed with contrast agent iohexol (Omnipaque 350 mg/ml, GE Healthcare, USA) injected in a peripheral vein at a rate of 4 ml/s and a dose of 1.5 ml/kg, with a total volume of approximately 60–80 ml. Axial CT images were used to draw a region of interest in the left atrium to compute a time versus contrast density curve. This curve was used to establish the delay between the start of contrast injection and the start of the DECT acquisition. The scans were acquired in static lung conditions, covering the whole lung parenchyma in supine position. To avoid streak artifacts due to highly concentrated contrast-mean in the superior vena cava territory, scans were acquired in the caudocranial direction. The dual-energy imaging protocol consisted of a collimation of 0.6 mm, as well as of a tube voltage of 80 or 100 kV and Sn140 kV for respective tube and a reference tube current of 190/95 mAs using automatic tube current modulation (CARE Dose, Siemens Healthineers, Erlangen, Germany). The mean CTDIvol was 15.7 ±7.0 mGy (see *Figures 1 and 2*). The quality of each collected DECT scan was confirmed by one of the authors (TH), radiologist specialized in thoracic radiology.

**Laboratory, clinical and physiological variables**

Comprehensive clinical data (e.g., demographics, chronic health conditions, vital signs, ventilatory settings and laboratory tests, including markers of acute inflammation were collected from the patients’ medical records. All patients were not vaccinated for SARS-CoV2. A subgroup analysis was conducted, dividing the sampled population in two groups: the group of patients not treated with steroids (No-Steroids group) and the one treated with steroids (Steroids group). All patients in the No-Steroids group were admitted before June 16th, 2020. In June 2020 the early report of the RECOVERY dexamethasone trial [10,18] was published and the treatment guidelines of the disease were changed accordingly. Consequently, all patients recruited after June 16th, 2020, were treated with steroids, forming up the group labeled “Steroids”. Patients chronically treated with steroids for reasons different from COVID-19 but that received, during their ICU stay, a steroids dose comparable to or higher than Dexamethasone 6mg/day were included in the Steroids group. The first arterial blood gas analysis collected after patient transportation to the CT lab was collected.

**DECT image analysis**

The sequences of raw DECT datasets obtained during the simultaneous rotation of the two x-rays tubes and the two corresponding detectors were stored and subsequently processed using the DECT post-processing software package (Syngo.via, version VB10B, Multimodality Workplace; Siemens Medical Solutions) which generated material-specific DECT maps by applying a three-material decomposition algorithm [3]. By this way, two different series of images were produced and used for further analysis: the gas-distribution (pulmonary gas volume) maps and the corresponding blood-distribution (pulmonary blood volume) maps (see *Figures 1 and 2*). Syngo.via obtains pulmonary gas volume maps by subtracting iodine from dual energy data set and pulmonary blood volume maps by applying known attenuation-range values for air, soft tissue, and iodine to calculate their relative contribution to each voxel and estimate the amount of blood at a regional level. The obtained sequences of pulmonary gas volume maps and blood volume maps were, then, stored in DICOM format and further processed as two-dimensional matrices in the MatLab environment (MatLab and Statistics Toolbox Release 2021a, The MathWorks, Natick, USA). Twenty images evenly spaced along the cranial-caudal axis and located between the apex (set as cranial limit) and the diaphragmatic dome (set as caudal limit) were selected for analysis (see *Figure 1 and 2*). For each image, a manual lung parenchyma segmentation was applied to extract information from the only lung parenchyma. Big vessels, heart and mediastinal structures were excluded from the subsequent analysis [1]. The collected images were two-dimensional square matrices (512 x 512 voxels) having as voxel dimensions of 0.7461 mm x 0.7461 mm x 1 mm. Each voxel was characterized by a CT number, expressed as Hounsfield units (HU).

As regard to the pulmonary gas volume maps, the Hounsfield units (HU) range between –1,000 (gas) and +100 (non-aerated lung) was considered, as done in previous studies [2,3] (see *Figure 2*). Consequently, based on previous literature [4], four lung compartments were defined: hyperinflated (between –1,000 and –800 HU), normoinflated (between –800 and –500 HU), poorly inflated (between –500 and –100 HU) and non-inflated or atelectatic lung (between –100 and +100 HU).

As regard to pulmonary blood volume maps, a HU range between -100 (corresponding to non-perfused lung) and +150 (corresponding to high perfused lung) was analyzed (see *Figure 2*). Based on previous studies [5,6], zero HU was defined as limit to separate non-perfused (HU lower or equal to 0) from perfused lung (HU higher than 0). To quantify the extension of each HU compartment, each area was separately computed and expressed as both cm^2^ and percent of the total area in a given pulmonary blood volume (or gas volume) map [7]. From the bell-shaped curves characterizing the HU distribution of perfused blood volume maps, kurtosis was calculated, as measure of both dispersion and morphology of the curves (see Figure 3A) as done in previous studies [8–11] (see *Figure 3A*). A reduced kurtosis corresponds to a lower homogeneity of pulmonary blood volume within the analyzed lung parenchyma. Based on previous evidences [12], a reduced homogeneity in regional pulmonary blood volume can be interpreted as hypoxic pulmonary vasoconstriction (HPV). To assess HPV, in which a reduced blood volume is mainly expected in the hypoinflated area compared to the whole lung parenchyma, pulmonary blood volume distribution was separately assessed for the whole lung and for the hypoinflated lung, the latter characterized by poorly inflated and not inflated lung regions (HU between -500 and +100) (see *Figure 3*).

**Lung weight analysis**

Based on previous literature [13–16], for each selected pulmonary gas volume map, lung volume (ml), gas volume (ml) and lung weight (g) were calculated according to the following equations:

1. Gas volume (ml) = slice thickness (mm) * voxel area (mm^2^) * voxel attenuation (HU) /-1000 (HU);
2. Lung weight (mg) = slice thickness (mm) * voxel area (mm^2^) * (1 - (voxel attenuation (HU)/-1000 (HU))).

Hence, the total lung volume (ml), the total gas volume (ml) and the total lung weight (g) were calculated as follow:

$$Mlung= \sum_{i=1}^{N-1} \left( f* \frac{M_{i}+M_{i+1}}{2*t} \right)+ \frac{M_{1}+M_{N}}{2}$$

Where *N* was the number of slices, *t* was the slice thickness, *f* the distance between slices and *Mi* the lung mass in the *i* slice. This equation was used for both total lung parenchyma and for each lung compartment. The latter were reported as absolute value as well as percent of the total lung parenchyma. The analysis of the lung weight was performed for the whole patients’ population as well as for the two studied subgroups: the No-Steroids and the Steroids group.

**Statistical analysis**

Data analysis and statistical tests were performed using dedicated MatLab scripts (MatLab and Statistics Toolbox Release 2021a, The MathWorks, Natick, USA). Continuous variables were reported as mean ± standard deviation (SD). All statistical tests were two sided; p < 0.05 was considered statistically significant. Being comparisons between two mutually exclusive groups of the same population, adjustments of the significance level alpha for multiple comparisons was not performed. The Wilcoxon signed rank test was chosen as nonparametric test for two populations when the observations were paired (all parenchyma vs hypoinflated lung parenchyma). The Wilcoxon rank sum test was used as a nonparametric test in case of two independent patients’ populations (Steroids vs No-Steroids group). Fisher’s exact test (𝛼 = 0.05) was used, instead, to test statistically significant differences in case of categorical variables.

**Supplementary Reference**

1. Pellegrini M, Gudmundsson M, Bencze R, Segelsjö M, Freden F, Rylander C, et al. Expiratory Resistances Prevent Expiratory Diaphragm Contraction, Flow Limitation, and Lung Collapse. Am J Respir Crit Care Med. 2020;201.

2. Gattinoni L, Pesenti A, Bombino M, Baglioni S, Rivolta M, Rossi F, et al. Relationships between lung computed tomographic density, gas exchange, and PEEP in acute respiratory failure. Anesthesiology. 1988;69:824–32.

3. Gattinoni L, Caironi P, Pelosi P, Goodman LR. State of the Art What Has Computed Tomography Taught Us about the Acute Respiratory Distress Syndrome ? 2001;164:1701–11.

4. Gattinoni L, Caironi P, Pelosi P, Goodman LR. State of the Art What Has Computed Tomography Taught Us about the Acute Respiratory Distress Syndrome ? 2001;164:1701–11.

5. Hoey ETD, Mirsadraee S, Pepke-Zaba J, Jenkins DP, Gopalan D, Screaton NJ. Dual-energy CT angiography for assessment of regional pulmonary perfusion in patients with chronic thromboembolic pulmonary hypertension: Initial experience. American Journal of Roentgenology. 2011;196:524–32.

6. Ball L, Robba C, Herrmann J, Gerard SE, Xin Y, Mandelli M, et al. Lung distribution of gas and blood volume in critically ill COVID-19 patients: a quantitative dual-energy computed tomography study. Critical Care. 2021;25:1–12.

7. Uhrig M, Simons D, Ganten MK, Hassel JC, Schlemmer HP. Histogram analysis of iodine maps from dual energy computed tomography for monitoring targeted therapy of melanoma patients. Future Oncol [Internet]. Future Oncol; 2015 [cited 2022 Jan 18];11:591–606. Available from: https://pubmed.ncbi.nlm.nih.gov/25686115/

8. Yamashiro T, Matsuoka S, Estépar RSJ, Bartholmai BJ, Diaz A, Ross JC, et al. Kurtosis and skewness of density histograms on inspiratory and expiratory CT scans in smokers. COPD [Internet]. COPD; 2011 [cited 2022 May 1];8:13–20. Available from: https://pubmed-ncbi-nlm-nih-gov.ezproxy.its.uu.se/21299474/

9. Mascalchi M, Camiciottoli G, Diciotti S. Lung densitometry: why, how and when. J Thorac Dis [Internet]. J Thorac Dis; 2017 [cited 2022 May 1];9:3319–45. Available from: https://pubmed-ncbi-nlm-nih-gov.ezproxy.its.uu.se/29221318/

10. Perchiazzi G, Rylander C, Derosa S, Pellegrini M, Pitagora L, Polieri D, et al. Regional distribution of lung compliance by image analysis of computed tomograms. Respir Physiol Neurobiol [Internet]. Respir Physiol Neurobiol; 2014 [cited 2022 May 1];201:60–70. Available from: https://pubmed-ncbi-nlm-nih-gov.ezproxy.its.uu.se/25026158/

11. Matsuoka S, Kurihara Y, Yagihashi K, Niimi H, Nakajima Y. Quantification of thin-section CT lung attenuation in acute pulmonary embolism: correlations with arterial blood gas levels and CT angiography. AJR Am J Roentgenol [Internet]. AJR Am J Roentgenol; 2006 [cited 2022 May 1];186:1272–9. Available from: https://pubmed-ncbi-nlm-nih-gov.ezproxy.its.uu.se/16632718/

12. Iyer KS, Newell JD, Jin D, Fuld MK, Saha PK, Hansdottir S, et al. Quantitative dual-energy computed tomography supports a vascular etiology of smoking-induced inflammatory lung disease. American Journal of Respiratory and Critical Care Medicine. 2016;193:652–61.

13. Reske A, Reske A, Gast H, Seiwerts M, Beda A, Gottschaldt U, et al. Extrapolation from ten sections can make CT-based quantification of lung aeration more practicable. Intensive Care Medicine. 2010;36:1836–44.

14. Ball L, Braune A, Corradi F, Brusasco C, Garlaschi A, Kiss T, et al. Ultra-low-dose sequential computed tomography for quantitative lung aeration assessment—a translational study. Intensive Care Medicine Experimental. 2017;5:19.

15. Rylander C, Högman M, Perchiazzi G, Magnusson a, Hedenstierna G. Oleic acid lung injury: a morphometric analysis using computed tomography. Acta Anaesthesiol Scand [Internet]. 2004 [cited 2014 Sep 24];48:1123–9. Available from: http://www.ncbi.nlm.nih.gov/pubmed/15352958

16. Pellegrini M, Larina A, Mourtos E, Frithiof R, Lipcsey M, Hultström M, et al. A quantitative analysis of extension and distribution of lung injury in COVID-19: a prospective study based on chest computed tomography. Crit Care [Internet]. Crit Care; 2021 [cited 2022 Jan 18];25:1–12. Available from: https://pubmed.ncbi.nlm.nih.gov/34348797/
