# Supplementary material for: Chest dual-energy CT to assess the effects of steroids on lung function in severe COVID-19 patients
Source: Crit Care. 2022 Oct 25;26:328. doi: 10.1186/s13054-022-04200-z (PMC9595078; doi:10.1186/s13054-022-04200-z)
Supplement: Supplementary file 2 — Additional file 2. Table E1. Descriptive statistics for HU distribution for pulmonary gas volume and blood volume maps. Whole population (n = 60). Comparison between the whole parenchyma and hypoinflated areas. Data were reported as mean ± standard deviation (SD) as well as percent. Wilcoxon rank-sum test was used to test statistical differences between the whole parenchyma and the hypoinflated one (α = 0.05); *: to mark statistically significant differences. Table E2. Descriptive statistics for HU distribution for pulmonary gas volume and blood volume maps. Separately for the No-Steroids group (n = 17, Panel A) and the Steroids group (n = 43, Panel A) Comparison between the whole parenchyma and hypoinflated areas (Panel A and B) as well as between No-Steroids and Steroids group (Panel C). Data were reported as mean ± standard deviation (SD) as well as percent. Wilcoxon rank-sum test was used to test statistical differences (α = 0.05); *: to mark statistically significant differences. Figure E1. Not Perfused Area % vs Kurtosis scatter plot in the No-Steroids (left) and the Steroids (right) group. Data reported for all the included patients, divided in No-Steroids (left) and Steroids (right), in the whole parenchyma (blue) and the hypo-/non-inflated lung (red). The stars represent the mean values for each of the four groups of data. The group treated with steroids (right /stars) has significantly higher values of kurtosis than patients in the No-Steroids group (left/stars). The Not Perfused parenchyma in the hypo/not inflated lung is larger in the No-Steroids group (left red) compared to the Steroids group (right red). No differences are detectable in the Whole lung parenchyma (blue) between Steroids and No-Steroids group. These findings can be interpreted as preserved hypoxic pulmonary vasoconstriction (HPV) in the No-Steroids group and jeopardized HPV in the Steroids group. Figure E2. Distribution of total lung weight in the two analyzed groups: N-Steroids (left) a [file 13054_2022_4200_MOESM2_ESM.pdf]

WHOLE POPULATION

| Gas Volume | WHOLE LUNG | | HYPO/NON-INFLATED LUNG | |
|---|---|---|---|---|
| | MEAN | SD | MEAN | SD |
| Hyperinflated area ($cm^2$) | 994 | 756 | x | x |
| Normoinflated area ($cm^2$) | 1305 | 496 | x | x |
| Poorly inflated area ($cm^2$) | 889 | 398 | 889 | 398 |
| Non-inflated area ($cm^2$) | 372 | 321 | 372 | 321 |
| Hyperinflated area % | 27 | 17 | x | x |
| Normoinflated area % | 37 | 11 | x | x |
| Poorly inflated area % | 26 | 11 | 74 | 15 |
| Non-inflated area % | 11 | 10 | 26 | 15 |

| Blood Volume | WHOLE LUNG | | HYPO/NON-INFLATED LUNG | | Wilcoxon rank sum test. Whole vs Hypoinflated |
|---|---|---|---|---|---|
| | MEAN | SD | MEAN | SD | p ($\alpha = 0.05$) |
| Non-perfused area ($cm^2$) | 461 | 286 | 176 | 150 | 0.74 |
| Perfused area ($cm^2$) | 3005 | 913 | 1030 | 479 | < 0.01 * |
| Non-Perfused area % | 14 | 8 | 15 | 11 | 0.06 |
| Perfused area % | 86 | 8 | 85 | 11 | 0.06 |
| Kurtosis | 2.99 | 0.81 | 2.47 | 1.11 | < 0.01 * |

Table E1

No-Steroids

| Gas Volume Maps | WHOLE LUNG MEAN | WHOLE LUNG SD | HYPO/NON-INFLATED LUNG MEAN | HYPO/NON-INFLATED LUNG SD |
|---|---|---|---|---|
| Hyperinflated area ($cm^2$) | 1120 | 703 | x | x |
| Normoinflated area ($cm^2$) | 1456 | 587 | x | x |
| Poorly inflated area ($cm^2$) | 960 | 356 | 960 | 356 |
| Non-inflated area ($cm^2$) | 483 | 297 | 483 | 297 |
| Hyperinflated area % | 27 | 13 | x | x |
| Normoinflated area % | 36 | 9 | x | x |
| Poorly inflated area % | 24 | 8 | 68 | 12 |
| Non-inflated area % | 13 | 8 | 32 | 12 |

| Blood Volume Maps | WHOLE LUNG MEAN | WHOLE LUNG SD | HYPO/NON-INFLATED LUNG MEAN | HYPO/NON-INFLATED LUNG SD | Wilcoxon rank sum test. Whole vs Hypoinflated p ($\alpha = 0.05$) | |
|---|---|---|---|---|---|---|
| Non-perfused area ($cm^2$) | 568 | 346 | 261 | 144 | 0.01 | * |
| Perfused area ($cm^2$) | 3380 | 1148 | 1125 | 418 | < 0.01 | * |
| Non-Perfused area % | 15 | 7 | 19 | 8 | < 0.01 | * |
| Perfused area % | 85 | 7 | 81 | 8 | < 0.01 | * |
| Kurtosis | 2.43 | 0.50 | 1.91 | 0.35 | < 0.01 | * |

**Panel A**

Steroids

| Gas Volume Maps | WHOLE LUNG MEAN | WHOLE LUNG SD | HYPO/NON-INFLATED LUNG MEAN | HYPO/NON-INFLATED LUNG SD |
|---|---|---|---|---|
| Hyperinflated area ($cm^2$) | 944 | 778 | x | x |
| Normoinflated area ($cm^2$) | 1245 | 448 | x | x |
| Poorly inflated area ($cm^2$) | 861 | 414 | 861 | 415 |
| Non-inflated area ($cm^2$) | 328 | 324 | 328 | 324 |
| Hyperinflated area % | 26 | 19 | x | x |
| Normoinflated area % | 37 | 11 | x | x |
| Poorly inflated area % | 26 | 12 | 75 | 15 |
| Non-inflated area % | 10 | 11 | 25 | 15 |

| Blood Volume Maps | WHOLE LUNG MEAN | WHOLE LUNG SD | HYPO/NON-INFLATED LUNG MEAN | HYPO/NON-INFLATED LUNG SD | Wilcoxon rank sum test. Whole vs Hypoinflated p ($\alpha = 0.05$) | |
|---|---|---|---|---|---|---|
| Non-perfused area ($cm^2$) | 419 | 250 | 143 | 140 | < 0.01 | * |
| Perfused area ($cm^2$) | 2857 | 767 | 993 | 500 | < 0.01 | * |
| Non-Perfused area % | 13 | 8 | 13 | 11 | 0.42 | |
| Perfused area % | 87 | 8 | 87 | 11 | < 0.01 | * |
| Kurtosis | 3.22 | 0.80 | 2.69 | 1.21 | < 0.01 | * |

**Panel B**

No-Steroids vs Steroids

| Gas Volume Maps | p ($\alpha = 0.05$) | |
|---|---|---|
| Hyperinflated area ($cm^2$) | 0.24 | |
| Normoinflated area ($cm^2$) | 0.23 | |
| Poorly inflated area ($cm^2$) | 0.30 | |
| Non-inflated area ($cm^2$) | 0.01 | * |

WHOLE LUNG

| Blood Volume Maps | p ($\alpha = 0.05$) | |
|---|---|---|
| Non-perfused area ($cm^2$) | 0.09 | |
| Perfused area ($cm^2$) | 0.09 | |
| Non-Perfused area % | 0.11 | |
| Perfused area % | 0.10 | |
| Kurtosis | < 0.01 | * |

HYPO/NON-INFLATED LUNG

| Blood Volume Maps | p ($\alpha = 0.05$) | |
|---|---|---|
| Non-perfused area ($cm^2$) | < 0.01 | * |
| Perfused area ($cm^2$) | 0.25 | |
| Non-Perfused area % | < 0.01 | * |
| Perfused area % | 0.25 | |
| Kurtosis | < 0.01 | * |

**Panel C**

Table E2

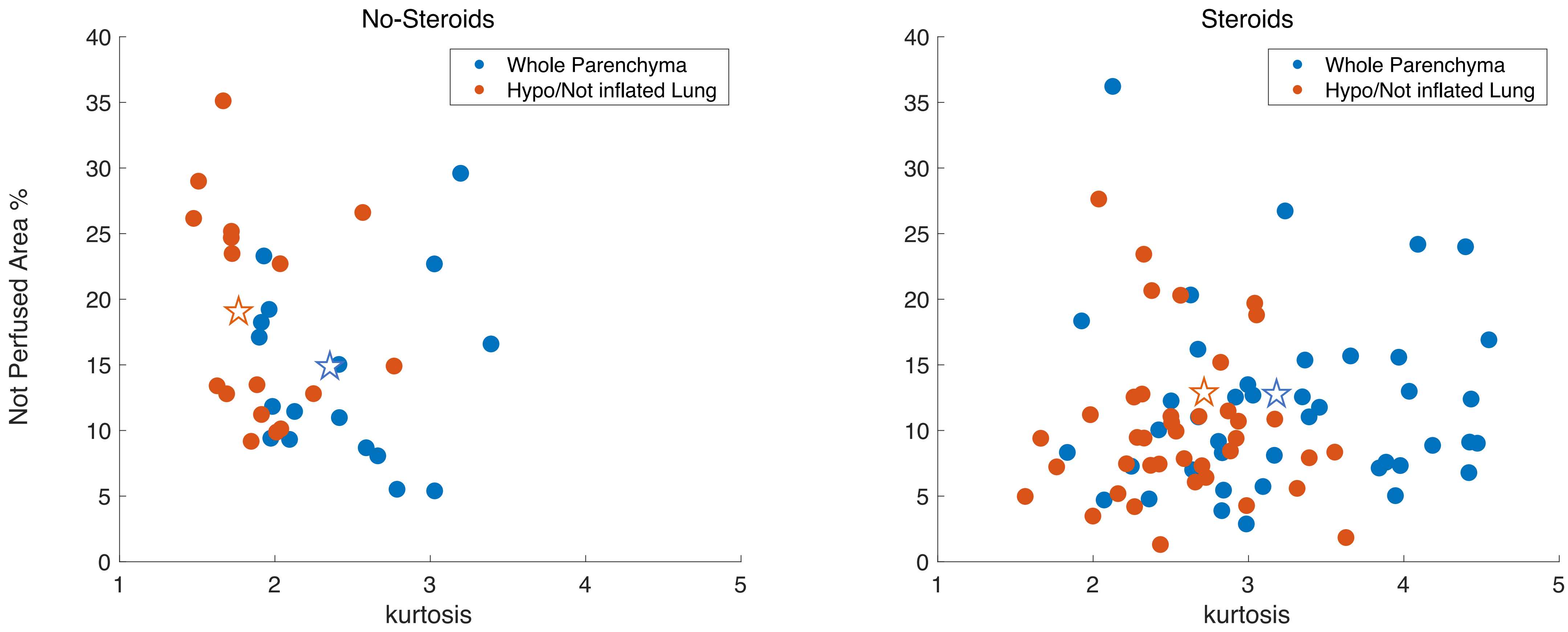

No-Steroids
Steroids
Whole Parenchyma
Hypo/Not inflated Lung
Not Perfused Area %
kurtosis
0
5
10
15
20
25
30
35
40
1
2
3
4
5


Figure E1

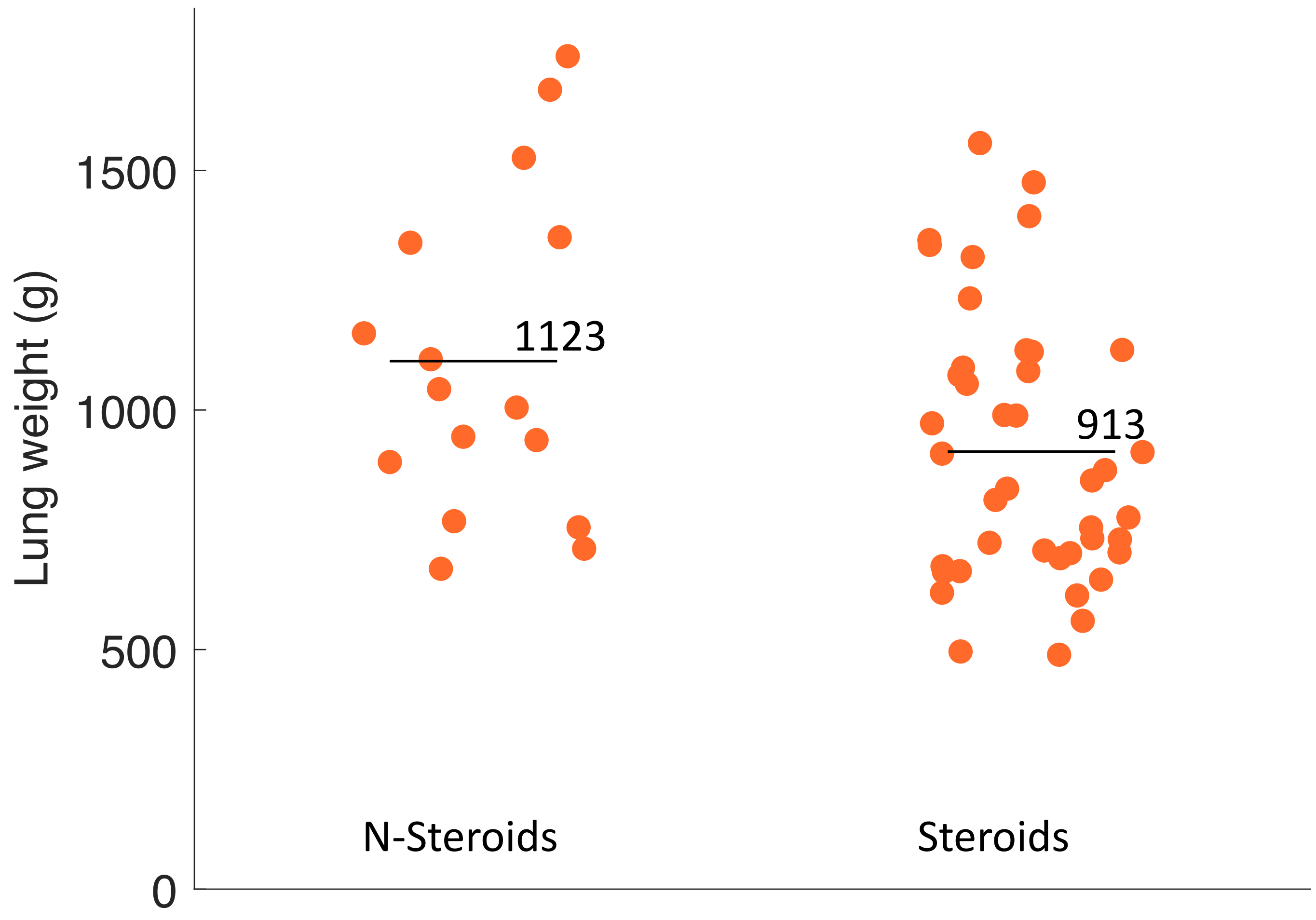


Figure E2
